# Genome-Wide Gene Expression Profiles in Antioxidant Pathways and Their Potential Sex Differences and Connections to Vitamin C in Mice

**DOI:** 10.3390/ijms140510042

**Published:** 2013-05-10

**Authors:** Yan Jiao, Hong Chen, Jian Yan, Lishi Wang, Yue Huang, Xiaoyun Liu, Robert W. Williams, Lu Lu, Yongjun Wang, Weikuan Gu

**Affiliations:** 1 Department of Orthopedic Surgery and BME-Campbell Clinic, University of Tennessee Health Science Center, Memphis, TN 38163, USA; E-Mails: yjiao2@uthsc.edu (Y.J.); jyan1@uthsc.edu (J.Y.); lwang37@uthsc.edu (L.W.); hyue@uthsc.edu (Y.H.); liuxyun6@mail2.sysu.edu.cn (X.L.); 2 Mudanjiang Medical College, Mudanjiang 157001, Heilongjiang, China; 3 The First Hospital of Qiqihaer City, 30 Gongyuan Road, Longsha District, Qiqihaer 161005, Heilongjiang, China; E-Mail: qsdyyych@yahoo.com; 4 Department of Anatomy and Neurobiology, University of Tennessee Health Science Center, Memphis, TN 38163, USA; E-Mails: rwilliams@uthsc.edu (R.W.W.); llu@uthsc.edu (L.L.); 5 Department of Neurology, Beijing Tiantan Hospital, Capital Medical University, Beijing 100050, China; E-Mail: yongjunwang1962@gmail.com

**Keywords:** L-*Gulo*nolactone oxidase, mouse, oxidative, vitamin C, sex

## Abstract

Vitamin C (VC) is well known as an antioxidant in humans, primates and guinea pigs. Studies have suggested gender differences in VC requirements in humans, and gender differences in oxidant injury vulnerability in early life may represent a biological mechanism contributing to gender disparity in later life. Using spontaneous bone fracture (*sfx)* mice, which lack the gene for L-*Gulo*nolactone oxidase (*Gulo*), we studied the potential sex difference in expression profiles of oxidative genes at the whole-genome level. Then, we analyzed data of gene expressions in a mouse population of recombinant inbred (RI) strains originally derived by crossing C57BL/6J (B6) and DBA/2J (D2) mice. Our data indicated that there were sex differences in the regulation of pre- and pro-oxidative genes in *sfx* mice. The associations of expression levels among *Gulo*, its partner genes and oxidative genes in the BXD (B6 × D2) RI strains showed a sex difference. Transcriptome mapping suggests that *Gulo* was regulated differently between female and male mice in BXD RI strains. Our study indicates the importance of investigating sex differences in *Gulo* and its oxidative function by using available mouse models.

## 1. Introduction

In humans, a gender difference in vitamin C (VC) requirement has been suggested by several studies. Levine *et al.*, investigated the association between dose and steady-state plasma concentration in young women and concluded that, while the recommended dietary allowance of VC for men is 75 mg daily, the recommended dietary allowance for young women should be increased to 90 mg daily [1]. Fain *et al.* [2] reported that gender is a risk factor for the hypovitaminosis C (plasma ascorbate <30 μmol/L) in hospitalized patients. Maruyama *et al.* [3] studied non-hospitalized 30- to 69-year-old Japanese to ascertain the influences of a 677C-T methylene-tetrahydrofolate reductase (MTHFR) genotype, nutritional intake and lifestyle-related factors on plasma homocysteine (Hcys) and serum folate concentrations. They found that log folate intake per 1,000 kcal in males was a significant and positive predictor of log serum folate concentration (*p* < 0.01), while in females, the log VC intake per standard body weight was a significant and positive variable (*p* < 0.001) predicting the log serum folate concentration. In a study on the association between the high VC intake and lower blood pressure levels and comparison between blood pressure and fruit and vegetable intake among German adults, Beitz *et al.* [4] found that, when information about VC and fruit and vegetable intake was considered simultaneously, a high fruit and vegetable intake was more strongly associated with lower systolic blood pressure levels, as compared with high VC intake among women. Interestingly, they did not find significant associations between blood pressure and vitamin C and fruit and vegetable intake among men. In a study on high cholesterol diet-induced renal injury in a rat model, Al-Rejaie *et al.* [5] concluded that high cholesterol diet-induced renal injury in female animals was higher than that in male animals, suggesting a better antioxidative stress defense response in males’ kidneys. Moreover, the antioxidant and renoprotective effects of rutin and VC were augmented following their combination.

VC is well known as an antioxidant in humans, primates and guinea pigs [6,7]. Oxidative stress is caused mainly by an imbalance between the activity of endogenous pro-oxidative enzymes and antioxidative enzymes in favor of the former [8]. Minghetti *et al.* [9] recently reported that sex-based differences in oxidant injury vulnerability occurring early in life could represent a biological mechanism contributing to gender disparity later in life. Antioxidative systems that protect from peroxidative damage are supposed to be under the influence of steroid hormones [10]. Genes in our study fit in those above-mentioned categories.

In spite of clear evidence for gender differences in VC metabolism and its connection to antioxidants in humans and sex difference in animals, the genetic bases and molecular regulations of those differences are not yet completely understood. During the past decade, we have been using a mouse model of spontaneous bone fracture (*sfx*) [11–14] to study the effects of VC on skeletal development. In an early study, we discovered that the *sfx* model lacks the gene for l-*Gulo*nolactone oxidase (*Gulo*), a key enzyme in the ascorbic acid (AA) synthesis pathway [12]. At the same time, in our study, we also take advantage of a unique resource of animal model, the BXD (C57BL/6J × DBA/2J) recombinant inbred (RI) strains [15]. The BXD strains are a well-characterized set of strains for which a remarkable variety of phenotypic data has already been acquired. Information on the phenotypes can be easily obtained from the GeneNetwork page: http://www.genenetwork.org. Gene expression profiles generated from livers of those RI strains are available for the analysis of molecular pathways [15]. Here, we report the potential difference in expression profiles of oxidative genes at the whole-genome level in *sfx* mice and in BXD RI mouse strains. Because the gender difference in VC requirement has been known and because of the known oxidative function of VC, we decided to investigate whether there is a sex difference in the effect of expression levels of oxidative-relevant genes because of a lack of VC and whether the effect on oxidative genes of VC is related hormones stimulation.

## 2. Results and Discussion

Our results are from two sets of experiments. The first set is from the comparison between the *sfx* mice and its wild-type control, the BALB/c mice. The second set is the pathway analysis from the BXD RI strains (derived from C57BL/6J and DBA/2J).

### 2.1. Expression Levels of Oxidative Genes between Female and Male in *sfx* Mice in Comparison to Its Wild-Type Control

In this section, we present the data from the comparison of levels of gene expression between male and female of *sfx* and BALB/c mice. The purpose of this part of the study is to examine if the expression levels of oxidative genes are different between male and female mice.

#### 2.1.1. Significant Changes in Pro-Oxidative Enzymes

**Figure 1:**
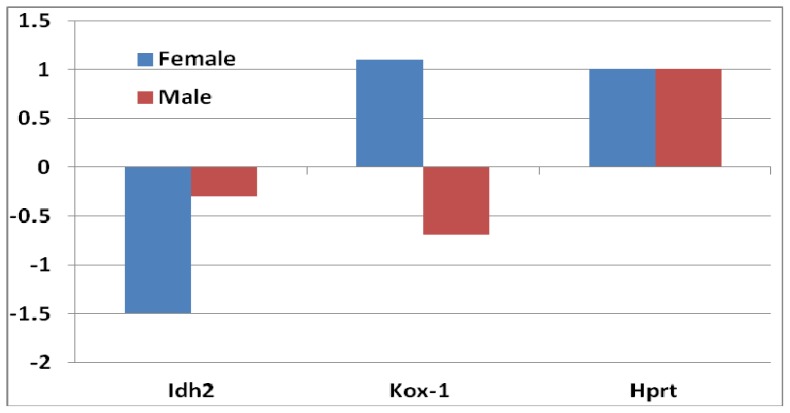
Fold changes of expression levels of three pro-oxidative genes in female and male mice between sfx and wild-type (WT) mice. *Y*-axis indicates the fold changes in comparison to WT BALB/c mice. The expression level of *Idh* is decreased in both female and male mice. The expression level of *Hprt* is increased in both female and male mice. The expression level of *Kox-1* in female is increased, while in males it is decreased, with a total difference of two-fold.

Mitochondria enzymes are important in the oxidative pathway. While most genes coding for mitochondria enzymes showed similar changes between female and male mice, changes in the expression level of isocitrate dehydrogenase 2 (NADP+), mitochondrial (*Idh2*), showed a difference between female and male mice (Figure 1). The downregulation of *Idh2* was greater in female than that in male mice. *Idh2* catalyzes oxidative decarboxylation of isocitrate to alpha-ketoglutarate, producing NADPH. Pro-oxidants induce oxidative stress, usually either by creating reactive oxygen species or inhibiting antioxidant systems.

We examined the expression of two pro-oxidative genes—kidney superoxide-producing NADPH oxidase (*Kox-1/Nox4*) and hypoxanthine guanine phosphoribosyl transferase (*Hprt*). While *Hprt* was upregulated in both female and male *sfx* mice, the expression of *Kox-1* was upregulated in females and downregulated in males (Figure 1). *Kox-1* functions as the catalytic subunit of the phagocyte NADPH oxidase (*phox*) [16]. *Kox-1* plays a crucial role in host defense, which is evident from recurrent and life-threatening infections that occur in patients with chronic granulomatous disease whose phagocytes genetically lack the superoxide-producing activity [17].

#### 2.1.2. Significant Changes in Antioxidative Enzymes

Cellular reactive oxygen species (ROS) production is increased by anticancer drugs. Ascorbic acid as an antioxidant is known to suppress ROS production. ROS, including superoxide anions and peroxides, induces oxidative stress. Our data showed that some ROS are differentially expressed in female and male *sfx* mice.

**Figure 2:**
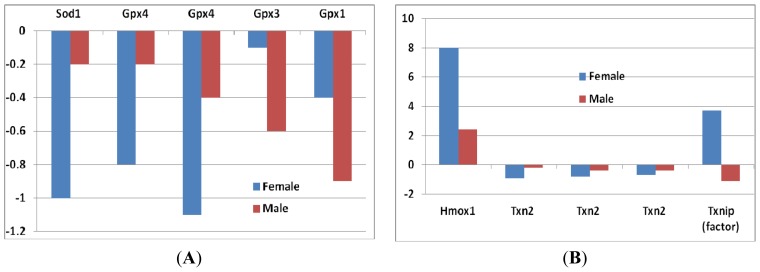

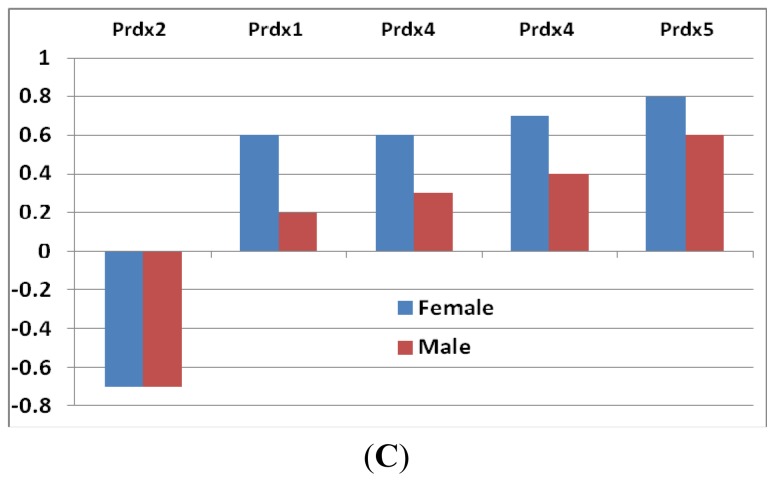
Fold changes of expression levels of antioxidative enzymes genes in female and male mice between *sfx* and WT mice. *Y*-axis indicates the fold changes in comparison to WT BALB/c mice. (**A**) The expression levels of superoxide dismutase 1 (*Sod1*) and glutathione peroxide (*Gpx1*, *Gpx3*, *Gpx4*); (**B**) The expression levels of Heme oxygenase and Thioredoxins in *sfx* mice; (**C**) The expression levels of peroxiredoxins in *sfx* mice. (**A**) (**B**)

1. *Superoxide dismutase 1 (Sod1):* In female mice, the decrease of *Sod1* expression was greater compared to that of male mice (Figure 2A), although it did not reach to a significant level.
2. *Glutathione peroxidase (Gpx):* Four probes of *Gpx*s suggested a decrease of *Gpx* genes in *sfx* mice. The decrease of *Gpx4* in female mice was greater than that in male mice, while the decrease of Gpx1 and Gpx3 in female was smaller than that in male mice (Figure 2A). None of those differences reaches to the significance level.
3. *Heme oxygenase:* The expression level of heme oxygenase (decycling) 1 (*Hmox1*) increased in both sexes, while the increase was much greater in females than that in males (Figure 2B). High levels of heme oxygenase-1 expression of cells can provide an antioxidant effect on skin, as well as anti-inflammatory properties, in mammals and rodents.
4. *Thioredoxin reductase (TrxR) and thioredoxin (Trx):* These enzymes are major regulators of intracellular protein thiol redox balance [18]. Their prolonged inhibition can disrupt a number of redox-sensitive functions in cells. Thioredoxin 2 (*Txn2*) was decreased in both sexes, but the decrease in females was greater than that in males. The expression level of thioredoxin interacting factor (*Txnip*) increased in females, while it decreased in males (Figure 2B).
5. *Peroxiredoxins:* These are important hydroperoxide detoxification enzymes, yet have only come to the fore in recent years relative to other major players in peroxide detoxification, heme-containing catalases and peroxidases and glutathione peroxidases [19]. Five family members of peroxiredoxin (*Prdx*) genes showed changes in expression levels (Figure 2C). *Prx2* showed a decrease in both sexes, while the other three (*Prdx1*, *Prdx4* and *Prdx5*) increased. The level of decrease in *Prdx2* was similar in both sexes. The increases of the other three *Prdx*s, however, were greater in females than that in males (Figure 2C).

#### 2.1.3. Sex Differential Expression of Genes Involved in Regulating Mapk Signaling

**Figure 3:**
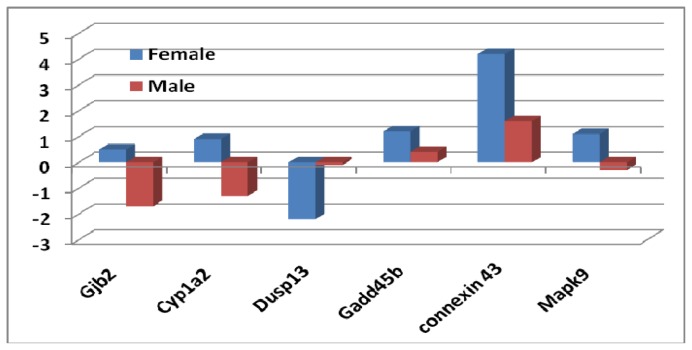
Fold changes of expression levels of genes involved in the regulation of Mapk signaling in female and male mice between *sfx* and WT mice. *Y*-axis indicates the fold changes in comparison to WT BALB/c mice.

Previously, we found that a group of genes related to a stress-activated protein kinase (*Sapks*) pathway was downregulated [13]. These genes included dual-specificity phosphatase, growth arrest- and DNA damage-inducible genes *gadd45*, beta (*Gadd45b*), *Connexin 43*, *Dusp13*, *Mapk9* (Jnk2) and *Cyp1A2*. We assume that the oxidative genes may be regulated by or connected to the genes in Mapk signaling. We therefore examined the expression of those relevant genes in both sexes (Figure 3). It appears that there is a sex difference in the expression of those genes.

#### 2.1.4. Gene Expression Levels of Growth Hormones in Female and in Male Mice between *Sfx* and WT Mice

**Figure 4:**
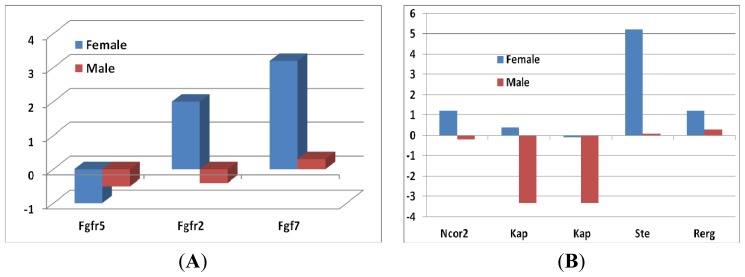
Fold changes of expression levels of growth hormones in female and male mice between *sfx* and WT mice. *Y*-axis indicates the fold changes in comparison to WT BALB/c mice. (**A**) The fold changes of the expression level of fibroblast growth factors in both female and in male mice; (**B**) The expression levels of sex hormones in *sfx* mice.

In our previous publication, we noted the significant effect of VC on hormone genes [13]. In this study, we found significant sex difference in hormone genes by comparing the effect on expression levels of hormone genes between female and male *sfx* mice. Figure 4 shows the effect of VC on growth hormones detected by expression level of probes of hormone genes.

Figure 4A shows the effect on genes of the androgen pathways, including fibroblast growth factor receptor 2 (*Fgfr2*), fibroblast growth factor receptor 5 (*Fgfr5*) and fibroblast growth factor 7 (*Fgf7*). The other set of hormones was the sex hormones, including silencing mediator of retinoic acid and thyroid hormone receptor alpha (*Ncor2*), kidney androgen-regulated protein (*Kap*), sulfotransferase, estrogen preferring (*Ste*) and RAS-like, estrogen-regulated, growth inhibitor (*Rerg*). We found a significant sex difference in the expression of *Ncor2* (Figure 4B).

While it was upregulated in females, it was unchanged in males. Two probes of *Kap* indicated a significant decrease in its expression in males. Both *Ste* and *Rerg* were upregulated in females, while they were unchanged in males.

#### 2.1.5. Validation of Microarray Data Using Real-Time qPCR

**Figure 5:**
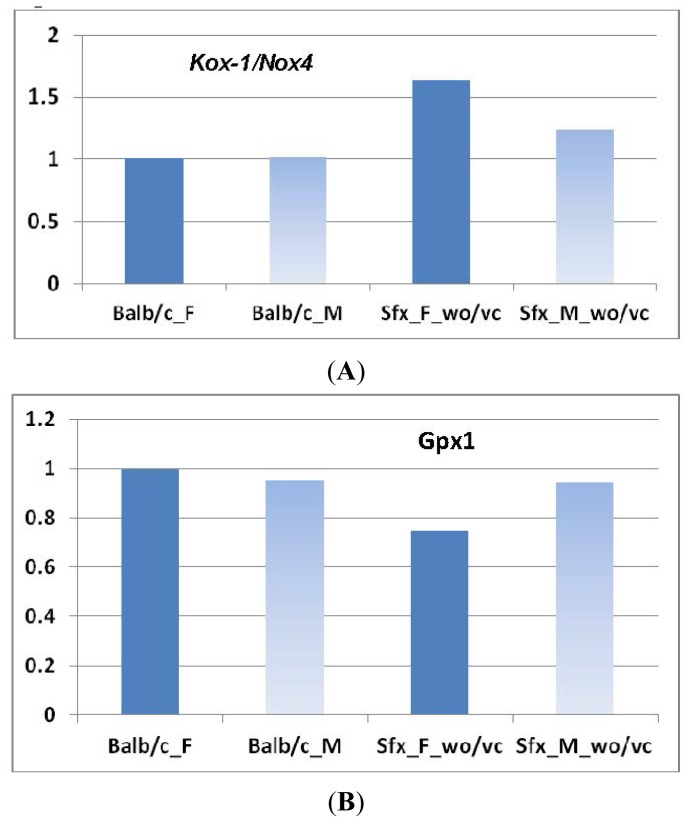
Gene expression levels measured by real-time qPCR of *Kox-1*/*Nox4* and *Gap1*of female and male mice between *sfx* and wild-type controls. *Y*-axis indicates the relative expression levels in comparison to WT female BALB/c mice, while the expression level of female BALB/c mice is given as one.

Although results from microarray have been regarded as the valid data for the gene expression in many studies, we conducted the real-time PCR on limited genes to conform our data of oxidative genes. We analyzed the expression of two key genes, *Kox-1*/*Nox4* and *Gpx1.* Results are shown in Figure 5. In microarray results*,* the expression of *Kox-1*/*Nox4* was upregulated in females and downregulated in males (Figure 1). The real-time PCR indicated that the expression in females is increased (*p* = 0.0099) and similar in males (*p* = 0.1535) (Figure 5A). In microarray, we showed the decrease of *Gap1* in both female and male *sfx* mice. In real-time PCR, the decrease in females reached a significant level (*p* = 0.0202), while in males it did not (*p* = 0.9384) (Figure 5B). These data again indicate that in general microarray and real-time PCR agree each other, while the degree of values differs.

### 2.2. Pathway Analysis Using Gene Expression Profiles of BXD Mice

In this section, we present the results of the analysis of gene network using whole genome gene expression profiles generated from livers of RI strains of BXD mice. The purpose of this portion of the study is to identify potential pathways that regulate the sex difference in oxidative genes.

#### 2.2.1. Gulo Gene and Its Partners

To identify a group of genes relevant to *Gulo* in normal mice, we input *Gulo* into GeneNetwork (http://www.genenetwork.org/webqtl/main.py) [20], which consists of a set of linked resources for systems genetics. We then obtained the interaction partner genes of *Gulo* based on STRING analysis.

**Figure 6:**
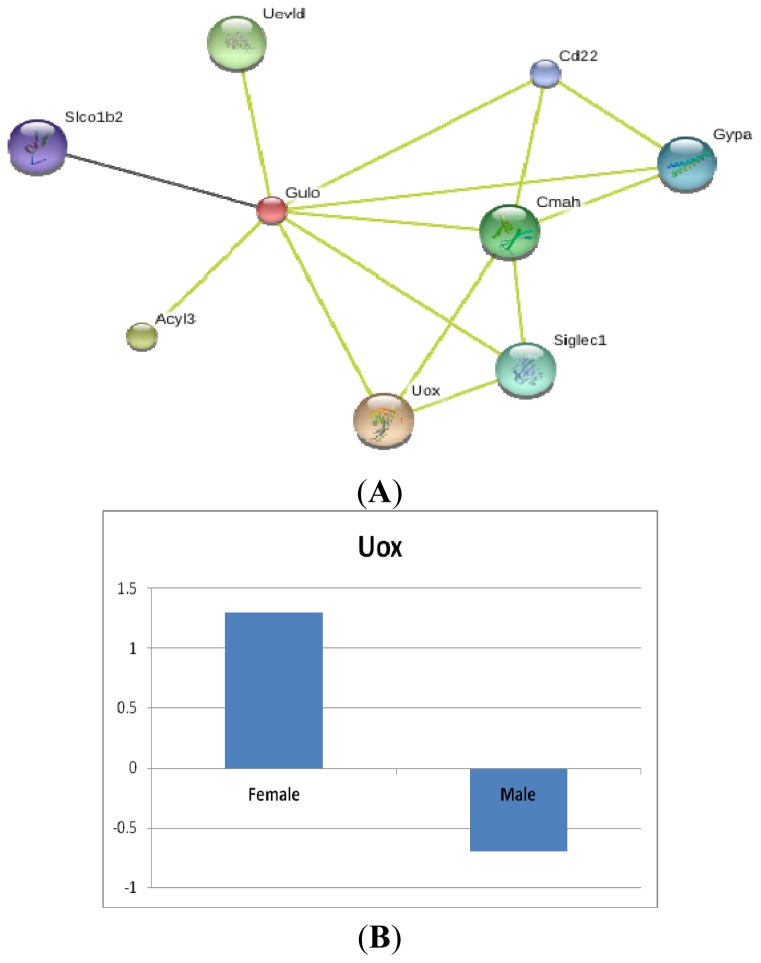
(**A**) Predicted interaction partner genes of *Gulo* based on STRING analysis. Different line colors represent the types of evidence for the association. Those genes include: *Uox*, urate oxidase gene; *Acyl3*, RIKEN cDNA 5330437I02 gene; *Uevld*, UEV and lactate/malate dehydrogenase domains gene; *Cmah*, cytidine monophospho-*N*-acetylneuraminic acid hydroxylase gene; *Siglec1*, sialic acid binding Ig-like lectin 1, sialoadhesin gene; *Gypa*, glycophorin A gene; *Cd22*, *CD22* antigen gene; *Slco1b2*, solute carrier organic anion transporter family, member 1b2 gene; (**B**) The differential expression of one of the *Gulo* partner, *Uox*, between *sfx* and WT control BALB/c mice.

As shown in Figure 6A, predicted functional partners included: (1) urate oxidase gene (*Uox*); in most mammals, the activity of urate oxidase catalyzes the oxidation of uric acid to allantoin; humans and some primates lack this enzyme activity. The loss of urate oxidase in humans during primate evolution predisposed humans to hyperuricemia, a metabolic disturbance that can lead to gouty arthritis and renal stones [21]; (2) *Acyl3* RIKEN cDNA 5330437I02 gene, a long-time lost gene in humans [22]; (3) UEV and lactate/malate dehydrogenase domains gene (*Uevld*), a possible negative regulator of polyubiquitination [23]; and (4) Cytidine monophospho-N-acetylneuraminic acid hydroxylase (*Cmah*). The expression of N-glycolylneuraminic acid (NeuGc) is controlled by cytidine monophospho-N-acetylneuraminic acid (CMP-NeuAc) hydroxylase activity, which in humans is inactivated by a deletion in the *CMAH* gene; (5) Sialic acid binding Ig-like lectin 1, sialoadhesin (*Siglec1*); two families of mammalian lectin-like adhesion molecules have been shown to bind glycoconjugate ligands in a sialic acid-dependent manner: the selectins and the sialoadhesins; (6) Glycophorin A (*Gypa*); *Gypa* is the major intrinsic membrane sialoglycoprotein of erythrocytes; (7) *CD22* antigen (*Cd22*) mediates B-cell/B-cell interactions; (8) Solute carrier organic anion transporter family, member 1b2 (*Slco1b2*) mediates the Na(+)-independent uptake.

Most of those genes are regarded as partners based on text mining of the literature, while *Slco1b2* is based on its coexpression with *Gulo*. None of these genes is among the oxidative genes in our analysis, except *Uox*, which appears to be a potential candidate in reactions with those oxidative genes and showed a sex difference (Figure 6B).

The possibility of oxidative pathways influenced by other mutations is small. Since the discovery of *sfx* mice, wild-type BALB/c mice have been used to cross to *sfx* in many generations. We therefore do not expect that the *sfx* mouse has the other mutation. As to the deletion region, we previously examined the genomic region of the deletion and did not find any other gene in the region [12].

#### 2.2.2. Transcriptomic Loci that Regulate *Gulo* in Female and Male Mice

**Figure 7:**
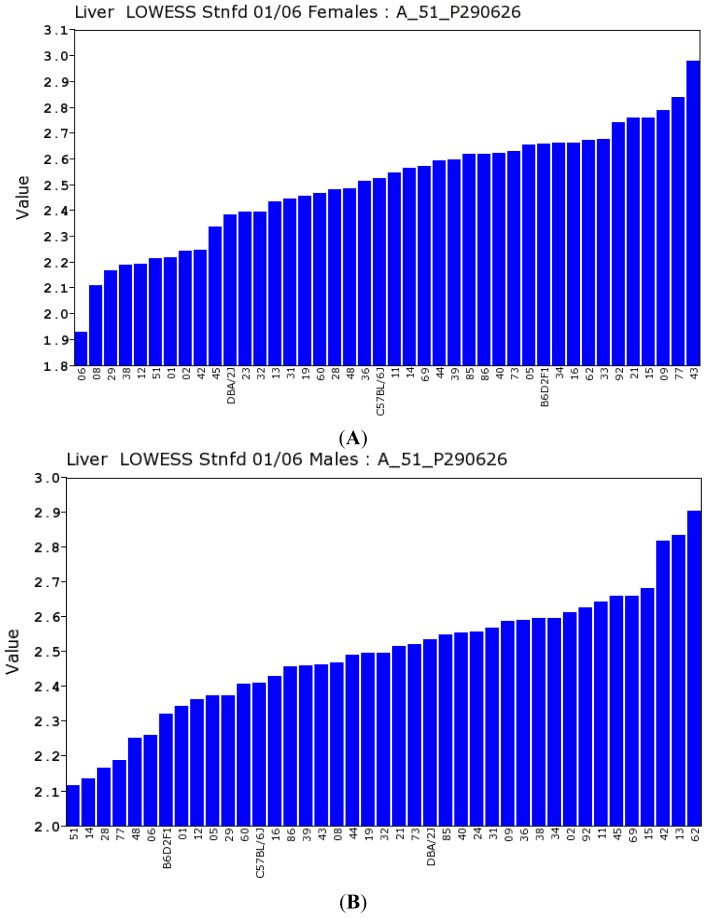
Expression levels of *Gulo* gene in the liver of parental strains and in recombinant inbred (RI) strains. *Y*-axis indicates the relative expression level of each strain. (**A**) Expression level of *Gulo* in female mice; (**B**) Expression level of *Gulo* in male mice.

There is a remarkable difference between the expression level of *Gulo* in female (Figure 7A) and male (Figure 7B) mice; we ranked the expression levels of *Gulo* low to high (Figure 7). The rank numbers of female and male mice D2 and B6 expression levels among the BXD strains are opposite from each other. In females, the rank numbers of D2 and B6 expression levels are 11th and 21st, respectively, and the F1 is 32nd. In males, the rank numbers of D2, B6 and F1 are at 24th, 13th and seventh, respectively.

**Figure 8:**
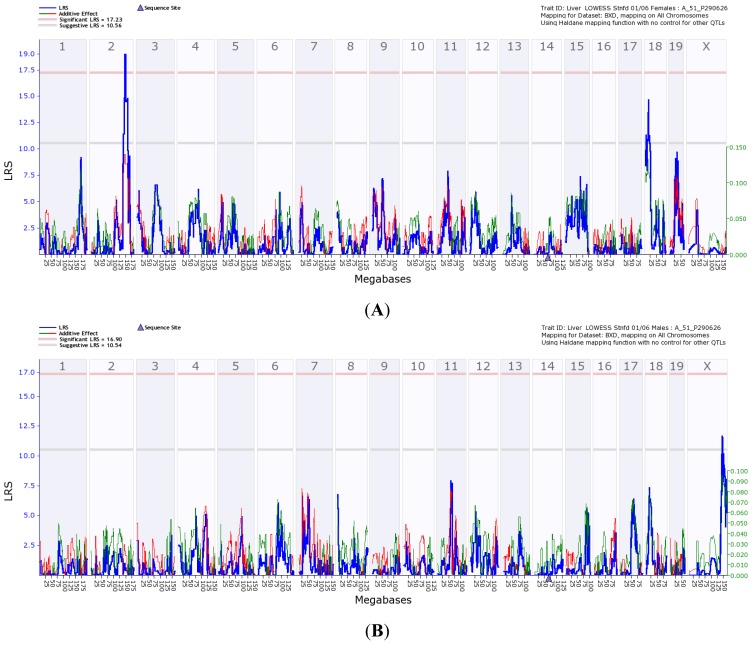

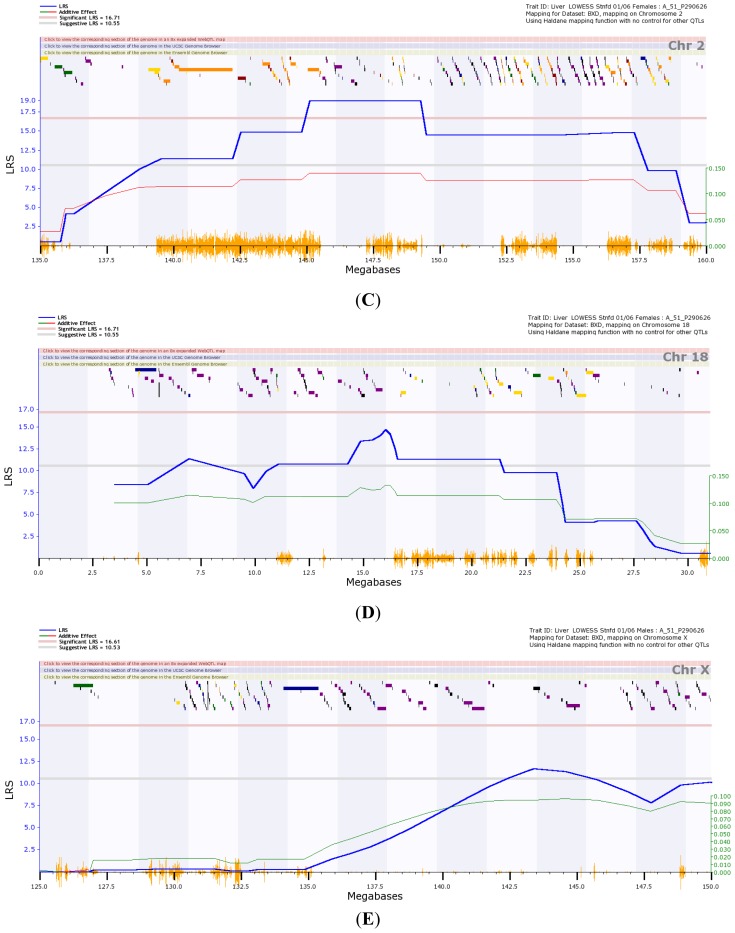
Transcriptome mapping of *Gulo* regulation Quantitative trait locus (QTL) based on gene expression profiles generated from livers of parental and BXD RI strains. The blue line ( 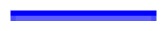) is the likelihood ratio statistic (LRS) score. The sold pink line ( 
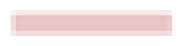
) indicates the level of significant LRS score, while the grey line ( 
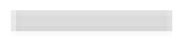
) indicates the suggestive LRS score. The green or red line ( 
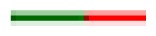
) is the additive score. (**A**) Transcriptome mapping of *Gulo* regulation QTL based on expression profiles generated from female strains, indicating that the two major loci located on Chr 2 and 18 regulate the expression of *Gulo*; (**B**) Transcriptome mapping of *Gulo* regulation QTL based on expression profiles generated from male strains, indicating that the major locus located on Chr X regulates the expression of *Gulo*; (**C**) The peak region of QTL on Chr 2 that regulates *Gulo* expression in female mice is located between 145 and 150 Mb; (**D**) The peak region of QTL on Chr 18 that regulates *Gulo* expression in female mice is located between 14 and 17 Mb; (**E**) The peak region of QTL on Chr X that regulates *Gulo* expression in male mice is located between 140 and 147 Mb.

Using the expression profiles of *Gulo* and the expression levels of whole genome genes in the liver of BXD strains, we generated the transcriptomic loci of *Gulo*. Transcriptome mapping using gene expression data from the livers of female and male mice led to identifying different loci involved in regulating *Gulo* gene expression (Figure 8).

The major transcriptome loci in females are located on chromosome 2 and 18 (Figure 8A), while the major regulator for *Gulo* in male mice is located on the X chromosome (Figure 8B). Examination of the genes in the peak region between 145 and 150 Mbp on Chr 2 identified 48 transcripts (Figure 8C), including 27 known genes. Investigation of the potential function of these genes identified two important oxidative-related genes, *Nkx2-2* and *Foxa2* [24], located in the critical region of the locus (Figure 8C). The QTL region on Chr 18 is similar to that we have reported previously [13]. Examination of the genes in the peak region between 14 and 17 Mbp on Chr 18 identified 18 transcripts (Figure 8D), including eight known genes. Investigation of the potential function of these genes identified *aquaporin 4* (*aqp4*) [25] as an important oxidative-related gene located in the critical region of the locus (Figure 8D). Examination of the genes in the peak region between 130 and 150 Mbp on Chr X identified 104 transcripts (Figure 8E), including 73 known genes. Investigation of the potential function of these genes identified five important oxidative-related genes: *Dcx*, *Trpc5*, *Wnk3*, *Apex2* and *Alas2* [26–28], located in the critical region of the locus (Figure 8E).

#### 2.2.3. Potential Gene Network Eluted from Whole-Genome Expression Profiles of Livers of BXD Strains

To see the associations of expression levels among those probes above from our analysis, we obtained the Spearman Rank Correlation (rho) using GeneNetwork, which found a total of 52 records. Those records included probes for *Alas2*, *Apex2*, *Aqp4*, *Cd22*, *Cmah*, *Cyp1a1*, *Cyp1a2*, *Dcx*, *Dusp13*, *Fgf7*, *Fgfr2*, *Fgfrl1*, *Foxa2*, *Gadd45b*, *Gja1*, *Gjb2*, *Gulo*, *Gypa*, *Hmox1*, *Hprt*, *Idh2*, *Kap*, *Kdap*, *Map3k5*, *Mapk9*, *Ncor2*, *Nkx2-2*, *Nkx2-3*, *Nkx2-4*, *Nkx2-5*, *Nkx2-6*, *Nkx2-9*, *Nox4*, *Pex5l*, *Prx1*, *Prrx1*, *Rerg*, *Shox2*, *Slco1b2*, *Sod1*, *Ste*, *Titf1*, *Trim28*, *Trip4*, *Trpc5*, *Txn2*, *Txnip* and *Uox*.

**Figure 9:**
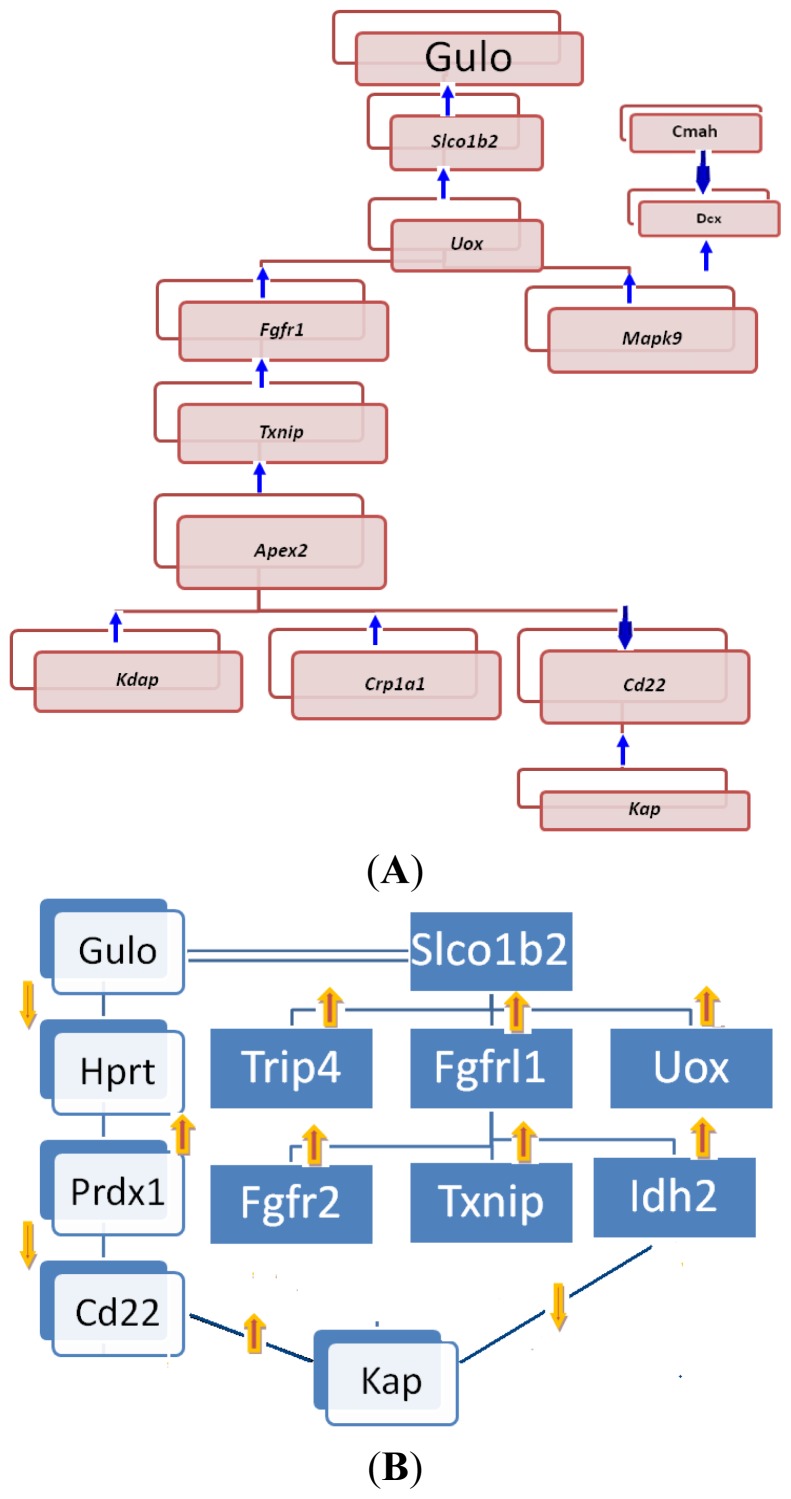
Potential pathways that *Gulo* regulate oxidative genes and other relevant genes based on known expression association of genes in livers from BXD RI strains and between *sfx* mice and its WT BALB/c. (**A**) Potential dominant pathways in female mice. Shadows behind the gene squares indicate that those pathways are female dominant, but males may also have them; (**B**) Potential dominant pathways in male mice. Those pathways are male dominant, but females may also have the similar pathways in addition to the female dominant pathways.

Based on the information in the rho table, we further examined the correlations between expression of *Gulo* and other genes. In females (Table S1), *Gulo* was directly positively correlated to *Slco1b2* (Figure 9A), while *Slco1b2* (Figure 9A) was positively correlated to *Uox* (Figure 9A). *Uox* was positively correlated to *Fgfrl1* (Figure 9A) and *Mapk9. Dcx* was positively correlated to *Mapk9; Cmah* was negatively correlated to *Dcx. Fgfrl1* was positively correlated to *Txnip* (Figure 9A). *Txnip* was positively correlated to *Apex2. Apex2* was positively correlated to *Kdap* and *Crp1a1* and negatively correlated to *Cd22. Cd22* was positively correlated to *Kap*. The positive interaction among *Gulo*, *Slco1b2* and *Uox* agreed with the results for *Gulo* and its partners obtained from STRING. The association in the expression among other genes has not been previously reported.

In males, the same number of records was found (Table S2). The associations, however, were different from those of female mice (Figure 9B). The expression of *Gulo* was negatively correlated to that of *Hprt*, which is on Chr X. *Hprt* was then positively correlated to Prdx1. Prdx1 was negatively correlated to *Cd22. Cd22* was positively correlated to *Kap; Slco1b2* was positively correlated to *Fgfrl1*, *Trip4* and *Uox. Fgfrl1* was positively correlated to *Fgfr2*, *Idh2* and *Txnip. Idh2* was negatively correlated to *Kap.*

Among those genes, the connection between *Fgfrl1* and *Fgfr2* was known. The associations of the expression of the other genes have not yet been documented.

The potential pathways in both sexes ended with *Kap*, which is regulated through different pathways in female and male mice. In females, *Kap* was negatively correlated to the expression of *Gulo* through the step of *Apex2* and *Cd22.* Thus, the deletion of *Gulo* in females did not affect the expression of *Kap*. These data agree with the data from *sfx* mice, in which the expression of *Kap* in females was not changed (Figure 4B). In males, however, two pathways regulate the expression of *Kap* (Figure 4B). There was a double negative correlation*, Gulo* to *Hprt* and Prdx1 to *Cd22*, between *Gulo* and *Kap*, thus leading to the positive correlation between *Gulo* and *Kap*. Therefore, the deletion of *Gulo* in *sfx* mice resulted in a downregulation of *Kap* in male mice (Figure 4B). In addition, there was a negative correlation between *Idh2* and *Kap* through the *Slco1b2* and *Fgfr2* axial, which was not directly correlated to the expression of *Gulo*. Note that the expression of *Fgfr2* (Figure 4A) and *Idh* (Figure 1) in male mice did not change much.

Based on the data from *sfx* and BXD mouse strains, we believe that there is a sex difference in the expression levels of oxidative genes. These mouse models are suitable for further investigation of molecular pathways in connection between oxidative genes and *Gulo*. The expression level of a number of oxidative genes showed sex differences in mice with or without deletion of *Gulo*, particularly *Fgfr2*, *Idh2*, *Txnip*, *Kap* and *Mapk9* [29–32]. Those results open the door for future studies. First, their potential roles in the connection between *Gulo* and oxidative genes can be further studied using these animal models. Second, confirmation of the gender differences of those oxidative genes in human populations may lead to different therapeutic applications for women and men. Gender-based treatment is an important part of individual medicine. We want to point out that those sex differences in pathways is at a relative level between two sexes; thus, the female and male pathways should actually be referred to as female and male dominant pathways. Similar pathways potentially exist for both sexes, while one is dominant over the other.

Our data indicated that the regulation of *Gulo* gene in mice is different between females and males. The transcriptome map suggests that, in females, the major loci that regulate *Gulo* are located on Chr 2 and 18, while in males, the major loci are on Chr X. The sex difference in expression of a number of oxidative genes in *sfx* mice suggests that *Gulo* potentially regulates oxidative pathways differently between females and males. The differences in growth and sex hormones and Mapk signaling suggest the possibility that the sex difference in the oxidative pathways is connected to hormones and Mapk signaling. Those genes showing a difference between female and male mice provide the basis for future investigation into the detailed regulation of oxidative pathways through *Gulo* gene. Those genes include the genes significantly changed in females and significantly downregulated in male mice. In *sfx* mice, the deletion of *Gulo* increased the expression of pro-oxidative genes, *Kox-1* in female mice. Four of five family members of peroxiredoxin (*Prx*) genes showed significant decrease in expression levels in female mice. Peroxiredoxins are important hydroperoxide detoxification enzymes, yet have only come to the fore in recent years relative to the other major players in peroxide detoxification, heme-containing catalases and peroxidases and glutathione peroxidases [19]. Evidently, the expression of several other relevant genes include *Txnip*, *Fgf7*, *Hmox1*, *Ste*, *Uox* and *Connexin 43* and are also increased in female mice. Several genes have significantly decreased expression levels in female mice: *Idh2*, *Sod1*, *Gpx4* and *Dusp13*. Those genes produce important enzymes/proteins to reduce the potential toxic effect of oxidative molecules. The protein product of *Idh2* catalyzes the oxidative decarboxylation of isocitrate to 2-oxoglutarate [33]. The protein product of *Sod1* binds to copper and zinc to degradate superoxide radicals and to prevent damage to organs and body [34]. *Gpx*4 is an essential antioxidant enzyme having multiple functions [35]. In male *sfx* mice, the expression of several genes showed a significant decrease: *Gjb2*, *Kap*, *Gpx*1 and *Gpx*3. It has been shown that *Kap* expression is critical for maintaining cardiovascular-renal homeostasis and that hypertension is associated with increased oxidative stress [36]. The expression and polymorphism of *Gpx*1 and *Gpx*3 have been linked to oxidative stress [37–39].

Many differentially expressed sex genes from *sfx* mice showed no associations to *Gulo* and its partners in the BXD strains. Many factors, including experimental errors, can lead to such a result. We believe that there are two main reasons. First, not every gene in the list from the *sfx* model has probes in the BXD analysis. We did not perform probes for 14 genes: *Ask1*, *Gpx1*, *Gpx3*, *Gpx4*, *Prx3*, *Prx4*, *Prx2*, *Prx5*, *Fgfr5*, *connexin43*, *Acyl3*, *Uevld*, *Siglec1* and *Wnk3*. Second, the effect of gene knockout is far more than that of the segregation of gene polymorphisms. The effect on genes in the whole genome by the *Gulo* gene in *sfx* mice is somehow manifested because of the deletion of *Gulo*. The affected genes include not only the genes in *Gulo*-related pathways, but also the genes in other pathways, which are affected by a gene in the *Gulo* pathways.

## 3. Materials and Methods

### 3.1. Animals

Two sets of animals were used. The first set was *sfx* mice and their wild-type. Homozygous *sfx*/*sfx* mice were bred at the animal facility at the Veterans Administration Medical Center (VAMC) in Memphis by vitamin C supplement. Wild-type BALB/c mice were purchased from The Jackson Laboratory and then housed at the VAMC. Three female and three male 6-week-old *sfx* mice were used for the experiment. Three age-matched female and three male WT mice were used as controls. The mice were handled according to a protocol previously described [12]. The 6-week-old mice were sacrificed, and femurs were immediately obtained and preserved in dry ice.

The second set of mice was the BXD mice. The BXD set of RI strains were derived by crossing C57BL/6J (B6) and DBA/2J (D2) and inbreeding progeny for 20 or more generations. The BXD mice were kept in Dr. Williams’ laboratory. All BXD strains were genotyped in the first half of 2005 at 13,377 markers as part of a CTC-Welcome Trust collaboration. Experimental procedures for this study were approved by the Institutional Animal Care and Use Committee at UTHSC and at VAMC at Memphis.

### 3.2. Procedure of Analysis of *sfx* Mice

Total RNA was isolated from the femurs of each sex (three WT and three *sfx* mice). Total RNAs were extracted from bones with Trizol Reagent [13] (Invitrogen, Carlsbad, CA, USA). Total RNA was purified by using the RNeasy MinElute Cleanup Kit (Qiagen, Valencia, CA, USA), and the quality of the total RNA was determined by Agilent Bioanalyzer 2100 (Agilent Technologies Santa Clara, CA, USA) The RNAs with an RNA Integrity Number (RIN) value greater than 8 were chosen for this study. The RNA was quantified by NanoDrop 2000 (Thermo Fisher Scientific, Waltham, MA, USA). Subsequently, 200 ng of high-quality RNA was used to generate cDNA and cRNA by using an Affymetrix GeneChip system with genome 430 2.0 arrays.

After *p-*value assessment (*p* < 0.05) [13,14], statistical analysis was done using EDGE software [15] to identify differentially expressed genes. Genes were notated as “present,” “absent,” or “may be present” according to Hayes *et al.* [15].

To validate the results from microarray, real-time qPCR of 2 genes, *Kox-1*/*Nox4* and *Gpx1*, in 4 groups of femur RNA samples, female wild-type, male wild-type, *sfx* female and sfx male mice, was conducted. Samples from 3 individuals of each group with duplicate wells were conducted by using ABI Prism 7500. The experimental procedure followed our previous publication [13].

### 3.3. Whole-Genome Expression Data of RI Strains of BXD Mice

Data of gene expression profiles in livers from BXD mice were obtained from Dr. Williams’s laboratory (http://www.genenetwork.org/webqtl/main.py). The data were produced by Agilent-011978 Mouse Microarray G4121A from 42 female and 41 male strains (see more information at http://www.genenetwork.org/dbdoc/LV_G_0106_F.html). The 42 female strains included two progenitors (B6, D2), An F1 and 39 BXD strains (BXD1, BXD9, BXD11, BXD12, BXD13, BXD14, BXD15, BXD16, BXD19, BXD2, BXD21, BXD23, BXD28, BXD29, BXD31, BXD32, BXD33, BXD34, BXD36, BXD38, BXD39, BXD40, BXD42, BXD43, BXD44, BXD45, BXD48, BXD5, BXD51, BXD6, BXD60, BXD62, BXD69, BXD73, BXD77, BXD8, BXD85, BXD86 and BXD92). The 41 male strains included two progenitors (B6, D2), An F1 and 38 BXD strains, most of which were the same as for female mice. Exceptions were BXD23 and BXD33, which were not included in male mice, while BXD24 was not included in female mice. Expression values were logged and then were further normalized and rescaled, so that the mean value for each array data set was 8 units, with a standard deviation of 2 units.

### 3.4. Transcriptome Mapping

Transcriptome mapping with GeneNetwork software was used to identify the chromosomal regions containing genes that affect the expression of *Gulo*. Gene expression data from the livers of the 42 female and 41 male strains were used in this analysis, which involved three major steps. First, *Gulo* was identified from the probes used to detect gene expression in BXD female and male RI strains. Second, interval mapping was done to establish *Gulo* transcriptome maps for the entire genome. Permutations of 2000 tests were used to assess the strength and consistency of the linkages. Third, partner genes for *Gulo* were determined by the STRING program.

### 3.5. Association of Expression Levels among Genes

Using Correlation Matrix, we analyzed the association of gene expressions. GeneNetwork provides tools to compute both Pearson product-moment correlations (the standard type of correlation) and Spearman rank order correlations. Analysis of association in expression between *Gulo* and relevant genes for oxidative reaction were conducted with whole-genome gene expression profiles of 42 female and 41 male mice of BXD strains. The genes were *Gulo*, *Idh2*, *Nox4*, *ASK1*, *Hprt*, *Sod1*, *Gpx1*, *Gpx3*, *Gpx4*, *Hmox1*, *Txn2*, *Txnip*, *Prx1*, *Prx3*, *Prx4*, *Prx2*, *Prx5*, *Fgf7*, *Fgfr2*, *Fgfr5*, *Gjb2*, *Cyp1a2*, *Dusp13*, *Gadd45b*, *connexin43*, *Mapk9*, *Kap*, *Ncor2*, *Trip4*, *Ste*, *RERG*, *Uox, Acyl3*, *Uevld, Cmah*, *Siglec1*, *Gypa*, *Cd22*, *Slco1b2*, *Nkx2-2*, *Foxa2*, *aqp4*, *Dcx*, *Trpc5*, *Wnk3*, *Apex2* and *Alas2.*

## 4. Conclusions

Our data suggest a sex difference in the expression levels of oxidative genes and the regulation of the *Gulo* gene and oxidative genes through *Gulo* in mice. There were sex differences in the regulation of oxidative genes by *Gulo* in *sfx* mice. The associations of expression levels among *Gulo*, its partner genes and oxidative genes in the BXD RI strains also showed a sex difference. Transcriptome mapping showed that *Gulo* was regulated on different chromosomal locations between female and male mice in BXD RI strains. It is important in the future to investigate sex differences in *Gulo* and its oxidative function by using available mouse models.
